# New Insight into the Octamer of TYMS Stabilized by Intermolecular Cys43-Disulfide

**DOI:** 10.3390/ijms19051393

**Published:** 2018-05-07

**Authors:** Dan Xie, Lulu Wang, Qi Xiao, Xiaoyan Wu, Lin Zhang, Qingkai Yang, Lina Wang

**Affiliations:** 1 Institute of Cancer Stem Cell, Dalian Medical University, 9 Western Lvshun South Road, Dalian 116044, China; danxie2016@hotmail.com (D.X.); xiaoqi1993a@hotmail.com (Q.X.); wuxiaoyan1234@hotmail.com (X.W.); zhanglindlmed@163.com (L.Z.); 2 School of Life Science and Biotechnology, Dalian University of Technology, No. 2 Linggong Road, Dalian 116024, China; wanglulu0813@126.com

**Keywords:** thymidylate synthase (TYMS), monomer, dimer, octamer, overexpression and purification

## Abstract

Thymidylate synthase (TYMS) is an essential enzyme for the de novo synthesis of deoxythymidine monophosphate (dTMP) and has been a primary target for cancer chemotherapy. Although the physical structure of TYMS and the molecular mechanisms of TYMS catalyzing the conversion of deoxyuridine monophosphate (dUMP) to dTMP have been the subject of thorough studies, its oligomeric structure remains unclear. Here, we show that human TYMS not only exists in dimer form but also as an octamer by intermolecular Cys43-disulfide formation. We optimized the expression conditions of recombinant human TYMS using the *Escherichia coli* system. Using high-performance liquid chromatography–tandem mass spectrometry (HPLC–MS/MS), we have shown that purified TYMS has catalytic activity for producing dTMP. In the absence of reductant β-mercaptoethanol, SDS-PAGE and size exclusion chromatography (SEC) showed that the size of the TYMS protein is about 35 kDa, 70 kDa, and 280 kDa. When the Cys43 was mutated to Gly, the band of ~280 kDa and the peak of the octamer disappeared. Therefore, TYMS was determined to form an octamer, depending on the presence of Cys43-disulfide. By measuring steady-state parameters for the monomer, dimer, and octamer, we found the k_cat_ of the octamer was increased slightly more than the monomer. On the basis of these findings, we suggest that the octamer in the active state might have a potential influence on the design of new drug targets.

## 1. Introduction

Classical thymidylate synthase (TYMS), encoded by the *thyA* gene, is highly conserved in most eukaryotes, including humans [1,2]. It catalyzes the transfer of a methylene group from the cofactor 5,10-methylenetetrahydrofolate (mTHF) to its substrate deoxyuridine monophosphate (dUMP) and forms deoxythymidine monophosphate (dTMP), yielding 7,8-dihydrofolate (DHF) as a secondary product [3,4,5]. A second class of thymidylate synthases, flavin-dependent thymidylate synthases (FDTSs) [6,7,8], is encoded by the *thyX* gene and has been found primarily in prokaryotes and viruses [6,9]. FDTSs utilize a noncovalently bound flavin adenine dinucleotide (FAD) prosthetic group to catalyze the redox chemistry and use mTHF only as a methylene donor. Several organisms, including human pathogens, rely solely on thyX for thymidylate synthesis. Recent studies further showed the catalytic mechanism of TYMS and FDTS [2,10], which are essential enzymes for DNA replication and frequently targeted by chemotherapeutic and antibiotic drugs [11,12]. However, drug resistance has become an increasing concern due to long-term use [13,14,15,16]. Therefore, researchers continue to search for effective and specific inhibitors of TYMS to overcome the resistance problem.

Extensive knowledge of the structure and properties of the target protein could contribute to formulating more efficient strategies for drug development. Many studies have reported that TYMS exists as a dimer–monomer equilibrium, whose two residues R175 and R176 form part of the dUMP binding site, and the TYMS dimer form can adopt active and inactive conformation [17,18,19]. There is evidence that the TYMS dimer interface plays an important role in TYMS–mRNA recognition, perhaps by controlling a conformational change of the protein that exposes the mRNA binding site [20,21,22]. In addition, Chu et al. thought the dimer obligates catalytic function, while the monomer is believed to play a crucial role in TYMS–mRNA regulation [23]. Considering these different insights in TYMS structure and function, it is of undeniable importance to further investigate the oligomeric form of the TYMS protein, which contributes to the design of compounds that bind at the oligomer interface of TYMS. Such compounds could overcome drug resistance problems [24]. 

The aim of this study was to determine the oligomeric state of TYMS and reconstitute the dTMP synthesis system in vitro. We optimized the overexpression conditions of TYMS, such as the host strain, the inducer concentration, temperature, and culture medium. TYMS catalytic activity for producing dTMP was assessed by mass spectrometry. More importantly, we used SDS-PAGE and size exclusion chromatography (SEC) to analyze the oligomeric state of TYMS. The data showed the full functionality of TYMS on DNA biosynthesis and demonstrated that TYMS coexists in an octamer–dimer–monomer equilibrium and that Cys43 disulfide contributes to octamer formation. In conclusion, our study demonstrated that the octamer exists in an active state by measuring steady-state parameters of different oligomeric form.

## 2. Results

### 2.1. TYMS Overexpression and Purification

To optimize the overexpression condition of the target protein, five different *Escherichia coli* strains (Tuner (DE3), BL21 (DE3), C41 (DE3), C43 (DE3), and BL21 (DE3)-pLysS) and bacteria concentrations with added isopropyl-β-d-thiogalactoside (IPTG) were initially used to screen. The results showed that 0.8 OD_600_ is optimal for pLysS (Figure S1A), C43 (Figure S1B), and C41 (Figure S1C), while 0.6 OD_600_ is optimal for BL21 (Figure S1D) and Tuner (Figure S1E). Then, comparing all of the expression levels of the optimal bacterial density of the different strains, we found 0.8 OD_600_ for pLysS is the optimal expression level (Figure S1F). Additionally, the concentration of IPTG, the temperature, and four different types of media were also screened. The optimal induction conditions for TYMS was found to be 0.4 mM IPTG and LB medium at 20 °C, after the cells reached 0.8 OD_600_ for pLysS (Figure S1G–I). Hence, we chose the recombinant 0.4 mM IPTG-LB-20 °C-0.8 OD_600_-pLysS for the large-scale expression condition.

After confirming the optimal system, the overexpressed TYMS was examined with Western blot and then purified with a nickel column twice. The samples were subjected to SDS-PAGE, followed by Coomassie brilliant blue staining after incubating at 100 °C with a loading buffer containing β-mercaptoethanol. Figure S2A shows that enrichment of TYMS via Ni-NTA chromatography yielded a large amount of full-length proteins. After the proteins were purified twice, they were diluted equivalently and subjected to SDS-PAGE followed by Coomassie brilliant blue staining (Figure S2B,C) to detect the purity of the proteins, which was found to be more than 90%. Finally, according to the BSA standard curve (Figure S2D), the concentration of purified TYMS was determined to be >60 mg/mL.

### 2.2. Reconstitution of TYMS-Mediated dTMP Synthesis

**Figure 1:**
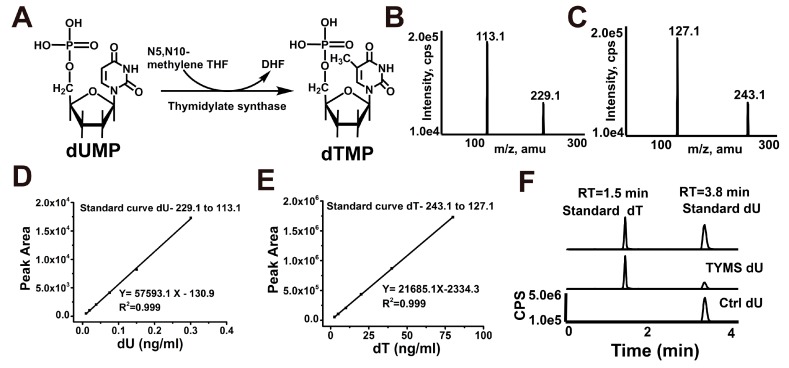
HPLC–MS/MS assay measures the reductive methylation activity of thymidylate synthase (TYMS) in vitro. (**A**) Deoxyuridine monophosphate (dUMP) and 5,10-methylenetetrahydrofolate (mTHF) as cosubstrates generate deoxythymidine monophosphate (dTMP) by TYMS, yielding 7,8-dihydrofolate (DHF) as a secondary product; (**B**,**C**) Base ion mass transitions for LC–MS-MS analysis of dU and dT standard. The multiple reaction monitoring (MRM) transitions were monitored as follows: 229.1 to 113.1 (dU); 243.1 to 127.1 (dT); (**D**,**E**) HPLC–MS-MS standards curves of dU and dT (F) LC–MS-MS profiles of nucleosides derived from TYMS treatment. The upper LC–MS-MS profile shows nucleoside dU and dT standards. The lower LC–MS-MS profile shows dT generation in vitro reaction system of TYMS with dU as substrate.

The catalytic mechanism of classical thymidylate synthases is presented in Figure 1A. To formally test the functionality of such a pathway and to provide a tool to investigate its mechanistic features, we reconstituted the entire process of reductive methylation with defined components. Using the dUMP substrate, we performed reductive methylation to measure the formation of dTMP using HPLC–MS/MS. Multiple reaction monitoring (MRM) was used to determine base ion mass transitions of dU (229.1 to 113.1) and dT (243.1 to 127.1) (Figure 1B,C). Standard curves were built to quantify dU and dT modification (Figure 1D,E). In the absence of TYMS, no dT was detectable. However, full reconstitution of the TYMS with the dUMP and mTHF substrates generated a substantial amount of dU (Figure 1F). Together, these results demonstrate that TYMS-mediated reductive methylation of dUMP generates dTMP. 

### 2.3. TYMS Formed Octamer by Intermolecular Cys43-Disulfide 

**Figure 2:**
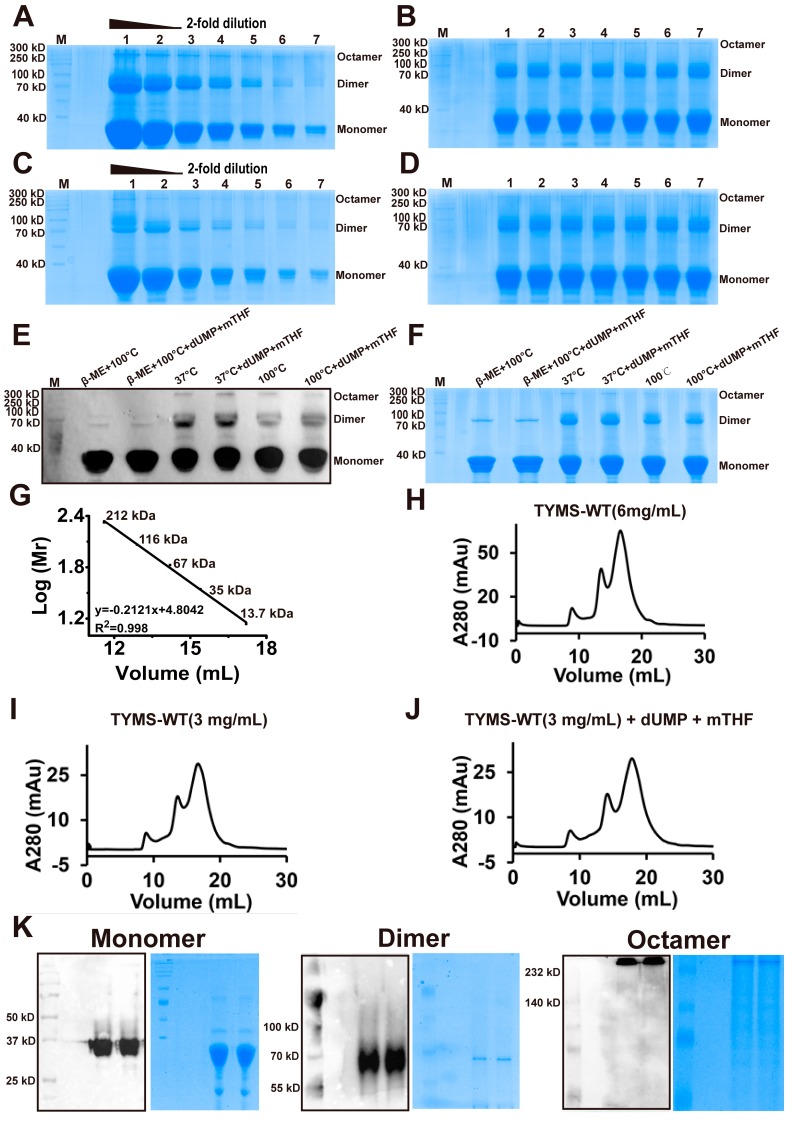
The determination of protein dimers and octamers. (**A**) Coomassie brilliant blue of concentrated proteins incubated without β-mercaptoethanol (β-ME) for 10 min at 37 °C: (1) undilutedly concentrated proteins; (2) double-diluted proteins; (3) 4-times diluted proteins; (4) 8-times diluted proteins; (5) 16-times diluted proteins; (6) 32-times diluted proteins; (7) 64-times diluted proteins. The MW of monomer, dimer, and octamer are ~35 kDa, ~70 kDa, and ~280 kDa, respectively. (**B**) Coomassie brilliant blue of eight diluted proteins incubated without β-mercaptoethanol (β-ME) for 10 min at 37 °C. (**C**) Coomassie brilliant blue of concentrated proteins incubated without β-mercaptoethanol (β-ME) for 10 min at 100 °C: (1) undilutedly concentrated proteins; (2) double-diluted proteins; (3) 4-times diluted proteins; (4) 8-times diluted proteins; (5) 16-times diluted proteins; (6) 32-times diluted proteins; (7) 64-times diluted proteins. (**D**) Coomassie brilliant blue of 8-times diluted proteins incubated without mercaptoethanol for 10 min at 100 °C. (**E**) Western blot and (**F**) Coomassie brilliant blue: (1) TYMS was directly incubated with a loading buffer (containing β-mercaptoethanol) for 10 min at 100 °C; (2) TYMS reacting with dUMP and mTHF for 1 h at 37 °C, and then incubated with a loading buffer (containing β-mercaptoethanol) for 10 min at 100 °C; (3) TYMS was directly incubated with a loading buffer (without β-mercaptoethanol) for 10 min at 37 °C; (4) TYMS reacting with dUMP and mTHF for 1 h at 37 °C, and then incubated with a loading buffer (without β-mercaptoethanol) 10 min in 37 °C; (5) TYMS was directly incubated with a loading buffer (without β-mercaptoethanol) for 10 min at 100 °C; (6) TYMS reacting with dUMP and mTHF for 1 h at 37 °C, and then incubated with a loading buffer (without β-mercaptoethanol) for 10 min at 100 °C. (**G**) Standard curve for molecular weight estimation and (**H**) size exclusion chromatography (SEC). Peak 1, 2, 3 represent a symmetrical peak eluted at a retention volume of ~8.9 mL, ~13.53 mL, and ~16.6 mL, indicating that TYMS existed in octamer, dimer, and monomer form. (**I**) Size exclusion chromatography of protein that was diluted to 3 mg/mL. (**J**) Size exclusion chromatography of protein that was diluted to 3 mg/mL with dUMP and mTHF. (**K**) The Western blot and Coomassie brilliant blue of components separated by SEC. The monomer was subjected to SDS-PAGE with 12% acrylamide, while the dimer and octamer were subjected to NATIVE-PAGE with 7.5% acrylamide.

To determine the existence of the homologous dimer, we used Coomassie brilliant blue and Western blot to analyze the status of the TYMS protein. Surprisingly, after TYMS incubated with a loading buffer (no β-mercaptoethanol) at 37 °C, we found that TYMS showed three bands with molecular weights ~35 kDa, ~70 kDa, and ~280 kDa, respectively (Figure 2A,B). Compared to the conditions at 37 °C, after TYMS incubated with a loading buffer (no β-mercaptoethanol) at 100 °C, the bands of ~70 kDa and ~280 kDa weakened (Figure 2C,D). While TYMS was incubated with a loading buffer (containing β-mercaptoethanol) at 100 °C, the band of ~280 kDa disappeared and the band of ~70 kDa weakened. This phenomenon made us speculate that TYMS may exist as an octamer; the proportion of each oligomeric form in different conditions are shown in Figure S3. To investigate the active or inactive conformation of TYMS, we incubated TYMS with dUMP and mTHF and subjected them to SDS-PAGE followed by Western blot (Figure 2E) and Coomassie brilliant blue (Figure 2F) staining. The results showed that the bands of ~70 kDa and~280 kDa remain unchanged, compared to samples without dUMP and mTHF. It is obvious from this result that the presence of ligands does not influence the oligomeric state of the enzyme.

To further investigate the oligomeric form of TYMS, we performed a calibration SEC experiment with five protein standards, including myosin (212.0 kDa), beta-galactosidase (116.0 kDa), bovine serum albumin (67.0 kDa), ovalbumin (43.0 kDa), and ribonuclease A (13.7 kDa). The established standard curve allowed a more reliable estimation of the protein molecular weight for the SEC column. As shown in the Figure 2G, the lg (Mr) value is plotted as a function of the retention volume. The experimental standard curve was well-fitted by the equation *y* = 2.46 − 0.016*x* with *R*^2^ = 0.996. TYMS (6 mg/mL) was subjected to SEC and collected at a retention volume of 8.9 mL, 13.53 mL, and 16.6 mL (Figure 2H). Meanwhile, TYMS (3 mg/mL) was subjected to SEC in the presence or absence of dUMP and mTHF (Figure 2I,J). The results indicated that the octamer is independent of the concentration and substrates of TYMS. Then, the collected samples were subjected to Western blot and Coomassie brilliant blue. (The Monomer was detected with SDS-PAGE (12% acrylamide), while the dimer and octamer were found with NATIVE-PAGE (7.5% acrylamide)) (Figure 2K). According to the standard curve equation and the Western Blot marker, the molecular weight of TYMS was estimated to be ~35 kDa, ~70 kDa, and ~280 kDa, which was about twice and 8-fold as much as the theoretical molecular weight of monomeric TYMS (35 kDa), indicating that the TYMS protein existed in oligomeric forms as a dimer and octamer by intermolecular Cys-disulfide.

**Figure 3:**
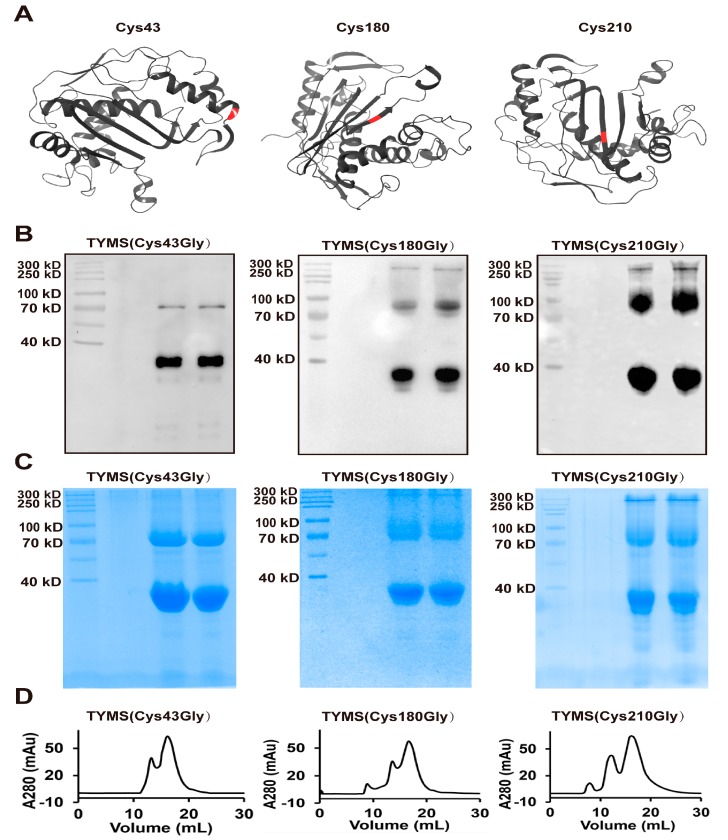
Determination of the oligomeric state of TYMS by mutated Cys. (**A**) Available TYMS structures with Cys43, Cys180, and Cys210 sites depicted as red. The data were derived from PDB (Protein Data Bank) and the ID is 1YPV; (**B**,**C**) Western blot and Coomassie brilliant blue of TYMS with Cys43Gly, Cys180Gly, or Cys210Gly (without β-mercaptoethanol); (**D**) size exclusion chromatography of TYMS with Cys43Gly, Cys180Gly, or Cys210Gly.

To determine which Cys site contributes to the form of the octamer, we examined the effect of Cys43, Cys180, and Cys210 disulfide on the TYMS octamer by mutating these to Gly (depicted as red in Figure 3A), due to Cys195 being an active site and Cys199 contributing to the dimerization interface [5,25,26]. TYMS (Cys43Gly), TYMS (Cys180Gly), and TYMS (Cys210Gly) were expressed and purified with Ni-NTA chromatography twice, and then they were detected by Western blot (Figure 3B), Coomassie brilliant blue without β-mercaptoethanol (Figure 3C), and SEC (Figure 3D). The band of octamer at ~280 kDa with a peak at 22.25 mL in TYMS (Cys43Gly) disappeared, while there were no changes in TYMS (Cys180Gly) and TYMS (Cys210Gly), which demonstrate the Cys43 residue is essential for the octamer. 

### 2.4. Analysis of Kinetic Properties of All Oligomeric Forms

**Figure 4:**
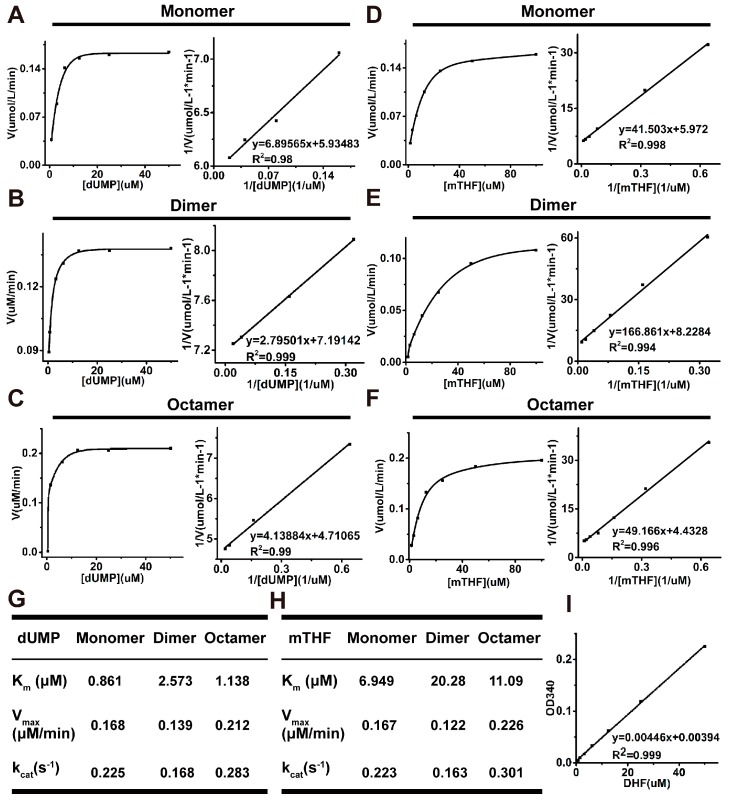
The reaction process curves and steady-state parameters for different oligomeric forms of TYMS. (**A**) Reaction process curves of dUMP for TYMS monomer, the left panel showing the substrate saturation curve, and the right panel showing the Lineweaver–Burk double-reciprocal plot; (**B**) reaction process curves of dUMP for TYMS dimer; (**C**) reaction process curves of dUMP for TYMS octamer; (**D**) reaction process curves of mTHF for TYMS monomer; (**E**) reaction process curves of mTHF for TYMS dimer; (**F**) reaction process curves of mTHF for TYMS octamer; (**G**) steady-state parameters of dUMP for different oligomeric forms; (**H**) steady-state parameters of mTHF for different oligomeric forms; (**I**) standard curve for ultraviolet absorption value at OD_340_ of DHF.

To further analyze the effect of the octamer, we detected the steady-state parameters of all the oligomeric forms by measuring the formation of 7,8-dihydrofolate (DHF). The reaction process curves for dUMP and mTHF of the monomer, dimer, and octamer are shown in Figure 4A–F, respectively. The K_m_ values for dUMP were increased by 3-fold for the dimer and 1.32-fold for the octamer compared with the monomer (Figure 4G). The K_m_ values for mTHF were increased by 2.9-fold for the dimer and 1.6-fold for the octamer, relative to for the monomer (Figure 4H). The V_max_ and k_cat_ values for dUMP of the dimer were decreased slightly, while the octamer increased more than the monomer, with the same results for mTHF, suggesting that the octamer of TYMS is in the active state. The experimental standard curve of DHF was well-fitted by the equation *y* = 0.00394 + 0.00446*x* with *R*^2^ = 0.999 (Figure 4I). HPLC–MS/MS was carried out to demonstrate the enzyme activity of the monomer, dimer, and octamer of TYMS (Figure S4).

## 3. Discussion

Recent research on the only de novo source of synthesized dTMP points towards a mechanism involving TYMS. Despite the classic nature of this pathway, experimental evidence that directly links TYMS activity with mTHF and dUMP is insufficient and the oligomeric form of TYMS remains unclear. This work aimed to analyze the oligomeric form of TYMS and reconstitute TYMS-mediated dTMP synthesis. 

In line with spectrophotometric assay [27], our work provides mass spectrum evidence for a direct productive action of TYMS with mTHF and dUMP, confirming dTMP generation (Figure 1). Reaction efficiency of dUMP to dTMP reaches at least 20%, suggesting the TYMS we purified has enzyme activity. Many studies have reported TYMS exists as the dimer, which has two distinct states: one is the active state in the crystal structures of TYMS-nucleotide-(anti) folate ternary complexes [28]; the other one is an inactive state in sulfate-containing conditions [29]. The cavity in the dimer interface could serve as an allosteric site used to regulate the conformational switching between the active and inactive states [1,30]. In addition, TYMS performs at least two different functions with specific interaction regions: the dimer obligates catalytic function, while both the monomer and the dimer are believed to play crucial roles in TYMS–mRNA recognition and regulation [31,32,33,34]. In this study, we found TYMS not only formed a dimer, but also an octamer by intermolecular Cys43-disulfide (Figure 3). The octamer is a higher homologous aggregation and in an active conformation, and the V_max_ and k_cat_ were increased slightly (<1.5-fold) for the octamer, while the K_m_ was decreased (<1.5-fold) (Figure 4). To the best of our knowledge, this is the first detailed report on the octamer of TYMS in an active conformation. Still, the role of the octamer is unclear. Since dimers have such an important function, it is possible that the octamer structure of TYMS may potentially affect the activity of TYMS so as to provide new drug targets to overcome resistance problems, as well as the synthesis of other nucleotides, so that the appropriate balance of the four nucleotides required for DNA synthesis is maintained. It should be noted that this study only examined the molecular weight of TYMS using SDS-PAGE and SEC. Our results lack crystallographic data. Despite its preliminary character, this study can clearly indicate TYMS exists in octamer–dimer–monomer equilibrium.

In summary, this work serves as the first comprehensive evaluation of the oligomeric structure and the activities of TYMS in vitro. Further, it provides a foundation for further inquiry into the role of this very interesting DNA synthesis and repair enzyme.

## 4. Materials and Methods

### 4.1. Plasmid Construction

The human TYMS (NM_001071.2) sequence was amplified by polymerase chain reaction (PCR) from human cDNA (reverse transcription from total mRNA) with primers (PET28A-TYMS-HindIII-Forward and PET28A-TYMS-XhoI-Reverse). TYMS (Cys43Gly), TYMS (Cys180Gly), and TYMS (Cys210Gly) were amplified by Fusion PCR [35] with primers PET28A-TYMS-HindIII-Forward, TYMS-43-Forward, TYMS-43-Reverse, TYMS-180-Forward, TYMS-180-Reverse, TYMS-210-Forward, TYMS-210-Reverse, and PET28A-TYMS-XhoI-Reverse (the sequence of the primers are shown in Table S1). The fragments were cloned into the pET-28a (+) vector using the restriction recognition site for *Hind* III and *Xho* I, carrying a N-terminal 6× His tag. DNA sequencing was used to verify the sequences of the constructed vector.

### 4.2. Expression Screening

To find out an optimized expression condition for TYMS, the vector pET-28a-6His-TYMS was transformed into Tuner (DE3), BL21 (DE3), C41 (DE3), C43 (DE3), BL21 (DE3)-pLysS. When IPTG (MedChemExpress, Monmouth, NJ, USA) was added, we optimized bacterial density, including concentrations of 0.3 OD_600_, 0.5 OD_600_, 0.8 OD_600_, 1.1 OD_600_, and 1.3 OD_600_, for different host strains. Furthermore, for the best host cell and its optional bacteria concentration, it was cultured in the nutrient-rich medium 32Y and several modified media, including LB, 2× TYE, and 4× TY (compositions of culture media are shown in Table S2) [36]. Then, the appropriate concentration of IPTG containing 0.1 mM, 0.2 mM, 0.4 mM, and 0.8 mM was investigated. Finally, the inducing temperature was optimized at 20 °C, 25 °C and 30 °C.

### 4.3. Protein Purification 

Cells were harvested by centrifugation at 5000× *g* for 10 min at 4 °C and washed twice with ice-cold PBS. Then, 2 g cell pellets were suspended with 10 mL of PBS, which contained 1 mM MgCl_2_, 10 mM imidazole (MedChemExpress), and protease inhibitor cocktail, and DNase at final concentrations of 1 mM, 20 mM, 1 mg/mL, 1 tablet/50 mL, and 100 U/mL, respectively. Resuspended cells were broken using an ultrasonic cell disruptor (NOISE ISOLATING CHAMBER JY 92-IIN) on ice (on 5 s, off 5 s). Lysed cells were subjected to centrifugation at 100,000× *g* for 1 h at 4 °C to obtain the supernatant. The supernatant containing TYMS was then loaded onto a Ni-NTA column pre-equilibrated with a binding buffer (PBS buffer, 150 mM NaCl, 10% (*v*/*v*) glycerol, 20 mM imidazole, pH 7.4). The resins were then washed eight times with the binding buffer containing 20 mM imidazole to remove nonspecifically bound proteins, and the bound proteins were eluted with an elution buffer (PBS buffer, 150 mM NaCl, 10% (*v*/*v*) glycerol, 400 mM imidazole, pH 7.4). To obtain more purified TYMS, we purified this protein twice using the Ni-NTA column as described above. Purified TYMS was concentrated using an Amicon Ultrafree centrifugal filter (Millipore Corporation, Billerica, MA, USA) with a cutoff of 10 kDa. The concentration buffer contained 20 mM Tris-Base, 150 mM NaCl, and 10% (*v*/*v*) glycerol at pH 7.4. Protein concentration was determined using the BCA assay according to manufacturer’s instructions (Pierce, Rockland, IL, USA). 

### 4.4. SDS-PAGE, Western Blot, and Coomassie Brilliant Analysis

After the purification, Coomassie brilliant blue was used to examine the purity of the proteins. Purified proteins were series diluted (0-, 2-, 4-, 8-, 16-, 32-, 64-, 128-, 256-fold) and then incubated with a loading buffer with or without β-mercaptoethanol at 37 °C or 100 °C for 10 min. Then, these sample were subjected to SDS-PAGE to determine the protein purity and oligomeric form. After that, 10 µM TYMS was incubated with 2 mM dUMP and 2 mM mTHF in a 40 µL reaction system at 37 °C and then subjected to SDS-PAGE to confirm the active or inactive state of TYMS.

Western blot was used to verify the protein expression, TYMSs were subjected to SDS-PAGE, the SDS gel was washed with a transfer buffer, and then the proteins were transferred from the gel onto a nitrocellulose membrane with a constant current of 250 mA for 2 h. The membrane was blocked with 1% nonfat milk powder in PBST (PBS containing 0.05% (*v*/*v*) Tween-20). Mouse anti-His-tag antibody (Sigma, St. Louis, MO, USA) was used as the primary antibody at a 1:5000 dilution in blocking solution. Goat-anti-mouse was the secondary antibody (Sigma), which was diluted at 1:3000, and protein bands were detected on photographic films using an enhanced chemiluminescent substrate.

### 4.5. Activity Detection

TYMS activity assays were carried out in 100 µL reaction volume containing a reaction buffer (10 mM boracic acid pH 6.0, 150 mM NaCl), 200 µM mTHF, 1 µM dUMP, and 10 µM TYMS. After incubation at 37 °C for 3 h, CIAP was added at a final concentration of 1 U and the reaction was incubated at 37 °C for 4 h. The samples were then subjected to HPLC–MS/MS analysis of deoxyuridine (dU) and deoxythymidine (dT). Quantification was performed using an HPLC system (Waters, Milford, MA, USA) coupled to an API 5500 triple quadrupole (ABSciex, Framingham, MA, USA) operating in positive electrospray ionization mode. The chromatographic separation was performed at 25 °C with the use of a C18 reverse-phase column (150 × 2.1 mm; 5 μm particle size; Thermo Fisher). The mobile phase consisted of A (water and 0.1% formic acid) and B (methanol and 0.1% formic acid) solutions [37]. The following conditions were employed during chromatography: 0.4 mL/min flow, 0–1 min, 1% B; 1–2 min, to 20% B; 2–3 min, to 20% B, 3–4 min, 1% B. To minimize potential salt and other contaminants in the ESI source, a time segment was set to direct the first 0.5 min of column elute to waste. For mass spectrometry detection, the multiple reaction monitoring was implemented using the following mass transitions: 243.1/127.1 (dT) and 243.1/127.1 (dU).

### 4.6. Size Exclusion Chromatography

Purified TYMS by Ni-NTA column was subjected to SEC at a flow rate of 1.0 mL/min on a Superdex-200 HiLoad 10/300 column (GE Healthcare, Pittsburgh, PA, USA) that had been pre-equilibrated with HEPES buffer (10 mM HEPES, 150 mM NaCl, 10% glycerol, pH 7.4). The eluent was collected in constant volumes of 500 μL and examined by ultraviolet absorption at 280 nm. Columns are often calibrated using five standard samples, including myosin (212.0 kDa), beta-galactosidase (116.0 kDa), bovine serum albumin (67.0 kDa), ovalbumin (43.0 kDa), and ribonuclease A (13.7 kDa).

The components separated by SEC were concentrated using an Amicon Ultrafree centrifugal filter (Millipore Corporation, Billerica, MA, USA) with a cutoff of 10 kDa in concentration buffer. The dimer and octamer separated by SEC were subjected to NATIVE-PAGE (7.5%) and the monomer was subjected to SDS-PAGE (12%), and then were analyzed by using Western blot and Coomassie brilliant blue. For NATIVE-PAGE, samples were run at constant voltage (100 V before the indicator to spacer gel and then switched to 120 V).

### 4.7. Reaction Kinetics Detection

Enzyme activity was determined by measuring the formation of dihydrofolate, which was monitored at 340 nm after the addition of the enzyme to the reaction assay [27,38]. Measurements were made at pH 6.0 and 37 °C in the reaction buffer (10 mM boric acid, 150 mM NaCl) for 5 min. To determine the K_m_(dUMP) for different oligomeric forms, varying concentrations of dUMP (0–50 µM) were used with constant concentrations of the enzyme (0.75 µM) and mTHF (100 µM). K_m_ (mTHF) was determined with varying concentrations of mTHF (0–100 µM) and were used with constant concentrations of the enzyme (0.75 µM) and dUMP (50 µM).

## Supplementary Materials

The supplementary materials are available online at http://www.mdpi.com/1422-0067/19/5/1393/s1.
