# Supplementary material for: New Insight into the Octamer of TYMS Stabilized by Intermolecular Cys43-Disulfide
Source: Int J Mol Sci. 2018 May 7;19(5):1393. doi: 10.3390/ijms19051393 (PMC5983622; doi:10.3390/ijms19051393)
Supplement: Supplementary file 1 [file ijms-19-01393-s001.pdf]

Supplementary Materials:

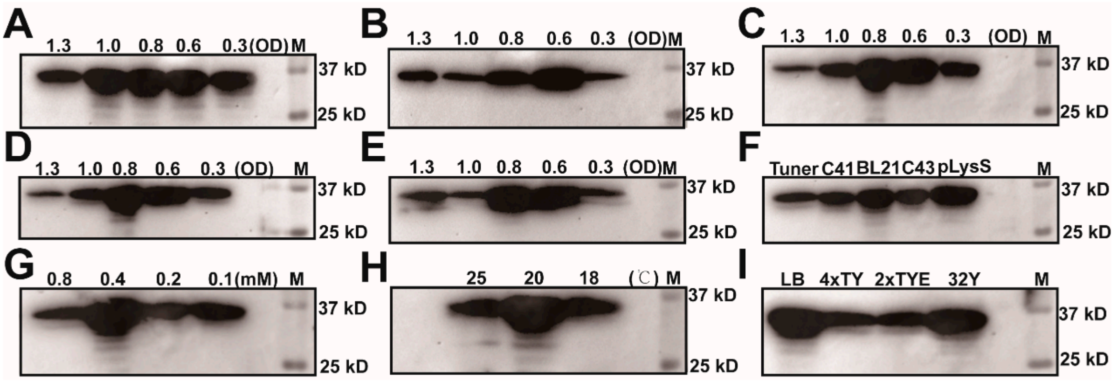

Figure S1. Screening of TYMS overexpression conditions.

(A) A concentration of 0.8 OD600 was found to be optimal for pLysS by comparing the expression level of different host strain concentrations by adding inducer IPTG. (B) A concentration of 0.8 OD600 was found to be optimal for C43 by comparing the expression level of different host strain concentrations by adding inducer IPTG. (C) A concentration of 0.8 OD600 was found to be optimal for C41 by comparing the expression level of different host strain concentrations by adding inducer IPTG. (D) A concentration of 0.6 OD600 was found to be optimal for BL21 by comparing the expression level of different host stain concentrations by adding inducer IPTG. (E) A concentration of 0.6 OD600 was found to be optimal for Tuner by comparing the expression level of different host strain concentrations by adding inducer IPTG. (F) pLysS was found to be the optimal host strain by comparing the expression level of other host strains with their best expression condition. (G) An optimal IPTG concentration of 0.4 mM was found for pLysS host strains when the cell concentration is 0.8 OD600. (H) The optimal temperature to induce protein expression was found to be 20 °C by comparing the expression level at 18 °C, 20 °C, and 25 °C. (I) LB is the optimal culture media, determined by comparing the expression level of four media (32Y, 2× TYE, 4× TY, and LB).

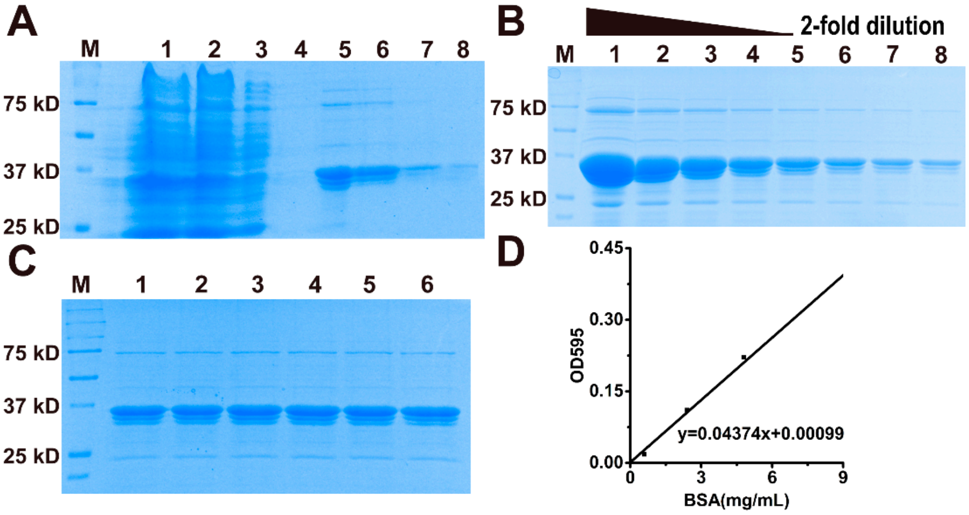

Figure S2. The purification of TYMS protein.

(A) Coomassie brilliant blue of the first purification by the nickel column: 1) unpurified sample; 2) filtrate of proteins after being combined with the nickel column; 3) filtrate of first washing with binding buffer; 4) filtrate of fifth time washing with binding buffer; 5) proteins collected from first eluting with elution buffer; 6) proteins collected from second eluting with elution buffer; 7) filtrate of first washing with elution buffer; 8) filtrate of second washing with elution buffer. (B) Coomassie

brilliant blue of second purification: 1) undiluted concentrated proteins; 2) double-diluted proteins; 3) 4-fold diluted proteins; 4) 8-fold diluted proteins; 5) 16-fold diluted proteins; 6) 32-fold diluted proteins; 7) 64-fold diluted proteins; 8) 128-fold diluted proteins. The band of ~35 kDa is the monomer of TYMS and the band of ~70 kDa is the dimer. (C) Coomassie brilliant blue of 8-times diluted proteins. (D) The proteins quantitative standard curve with BSA.

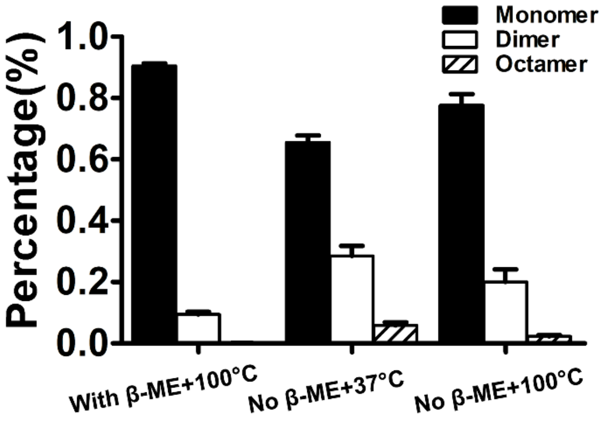

Figure S3. The proportion of each oligomeric form in different conditions.

When TYMS was incubated with a loading buffer (containing β-mercaptoethanol) at 100 °C, the proportions of the monomer, dimer, and octamer were 90.4%, 9.5%, and 0.1%, respectively. The proportions of each species were 65.5%, 28.6%, and 5.9% when the protein was incubated with a loading buffer (no β-mercaptoethanol) at 37 °C. When TYMS was incubated with a loading buffer (no β-mercaptoethanol) at 100 °C, the proportions of each species were 77.6%, 20.1%, and 2.3%.

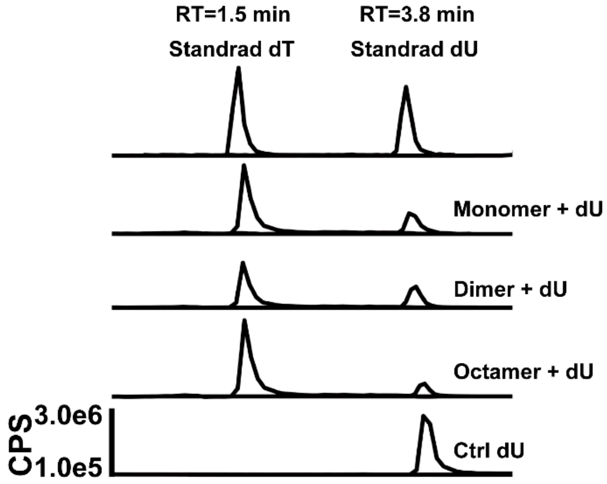

Figure S4. LC– MS-MS profiles of nucleosides derived from all oligomeric forms treatment.

The upper LC– MS-MS profile shows nucleoside dU and dT standards. The lower LC– MS-MS profile shows dT generation in vitro reaction system of all species with dU as substrate.

Table. S1 Sequence of primers

Name	Sequence
PET28A-TYMS-HindIII-Forward	CCAAGCTTGCATGCCTGTGGCCGGC
PET28A-TYMS-XhoI-Reverse	CCCTCGAGCTAAACAGCCATTTCCATTTTAAT
TYMS-43-Forward	CTCCGCGGCGGCGTCAGGAAGGACGAC
TYMS-43-Reverse	TTCCTGACGCCGCCGCGGAGGATGTGTTGGATCT
TYMS-180-Forward	ATCATCATGGGCGCTTGGAATCCAAGAG
TYMS-180-Reverse	TTCCAAGCGCCCATGATGATTCTTCTGTCGT
TYMS-210-Forward	TGAGCTGTCCGGCCAGCTGTACCAGAGATCG
TYMS-210-Reverse	ACAGCTGGCCGGACAGCTCACTGTTCACC

Table. S2 Compositions of culture media

Number	Medium	Medium component
1	32Y	3.2% (w/v) yeast extract, 0.8% (w/v) peptone, and 0.58% (w/v) NaCl in 10 mM Tris-HCl, pH 7.6
2	LB	5% (w/v) yeast extract, 10% (w/v) peptone, and 10% (w/v) NaCl, pH 7.6
3	2× TYE	1% (w/v) yeast extract, 1.6% (w/v) peptone, 0.5% (w/v) glucose, 0.5% (w/v) NaCl, 2 mM MgSO4, 20 mM KH2PO4, 80 mM K2HPO4
4	4× TY	2% (w/v) yeast extract, 0.8% (w/v) peptone, and 0.58% (w/v) NaCl
